# SARS-CoV-2 Delta and Omicron variants resist spike cleavage by human airway trypsin-like protease

**DOI:** 10.1172/JCI174304

**Published:** 2024-09-17

**Authors:** Wenyan Ren, Weiqi Hong, Jingyun Yang, Jun Zou, Li Chen, Yanan Zhou, Hong Lei, Aqu Alu, Haiying Que, Yanqiu Gong, Zhenfei Bi, Cai He, Minyang Fu, Dandan Peng, Yun Yang, Wenhai Yu, Cong Tang, Qing Huang, Mengli Yang, Bai Li, Jingmei Li, Junbin Wang, Xuelei Ma, Hongbo Hu, Wei Cheng, Haohao Dong, Jian Lei, Lu Chen, Xikun Zhou, Jiong Li, Wei Wang, Guangwen Lu, Guobo Shen, Li Yang, Jinliang Yang, Zhenling Wang, Guowen Jia, Zhaoming Su, Bin Shao, Hanpei Miao, Johnson Yiu-Nam Lau, Yuquan Wei, Kang Zhang, Lunzhi Dai, Shuaiyao Lu, Xiawei Wei

**Affiliations:** 1 Laboratory of Aging Research and Cancer Drug Target, State Key Laboratory of Biotherapy and Cancer Center, National Clinical Research Center for Geriatrics, West China Hospital, Sichuan University, Chengdu, Sichuan, China.; 2 National Kunming High-level Biosafety Primate Research Center, Institute of Medical Biology, Chinese Academy of Medical Sciences and Peking Union Medical College, Kunming, Yunnan, China.; 3 State Key Laboratory of Oral Diseases & National Clinical Research Center for Oral Diseases, West China Hospital of Stomatology, Sichuan University, Chengdu, Sichuan, China.; 4 Affiliated Dongguan Hospital, Southern Medical University (Dongguan People’s Hospital), Guangzhou, Guangdong, China.; 5 Department of Biology and School of Chinese Medicine, Hong Kong Baptist University, Kowloon Tong, Kowloon, Hong Kong, China.; 6 National Clinical Eye Research Center, Eye Hospital, Wenzhou Medical University, Wenzhou, Zhejiang, China.; 7 Institute of AI in Medicine and Faculty of Medicine, Macau University of Science and Technology, Taipa, Macau, China.

**Keywords:** COVID-19, Molecular biology

## Abstract

Soluble host factors in the upper respiratory tract can serve as the first line of defense against SARS-CoV-2 infection. In this study, we described the identification and function of a human airway trypsin–like protease (HAT), capable of reducing the infectivity of ancestral SARS-CoV-2. Further, in mouse models, HAT analogue expression was upregulated by SARS-CoV-2 infection. The antiviral activity of HAT functioned through the cleavage of the SARS-CoV-2 spike glycoprotein at R682. This cleavage resulted in inhibition of the attachment of ancestral spike proteins to host cells, which inhibited the cell-cell membrane fusion process. Importantly, exogenous addition of HAT notably reduced the infectivity of ancestral SARS-CoV-2 in vivo. However, HAT was ineffective against the Delta variant and most circulating Omicron variants, including the BQ.1.1 and XBB.1.5 subvariants. We demonstrate that the P681R mutation in Delta and P681H mutation in the Omicron variants, adjacent to the R682 cleavage site, contributed to HAT resistance. Our study reports what we believe to be a novel soluble defense factor against SARS-CoV-2 and resistance of its actions in the Delta and Omicron variants.

## Abstract 2

Mutations of P681R in Delta and P681H in Omicron variants confer resistance to the antiviral effects of human trypsin-like protease in the respiratory tract.

## Graphical abstract

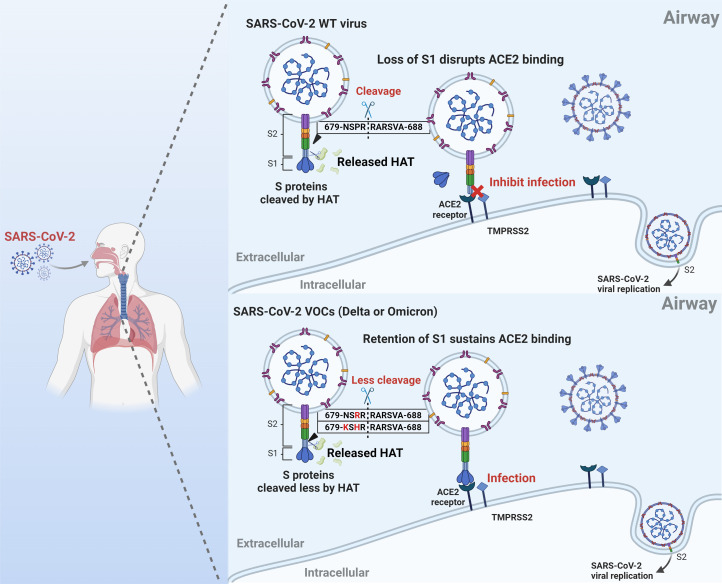

## Introduction

The causative agent for COVID-19, SARS-CoV-2, is a single-stranded, positive-sense RNA virus that replicates through the viral RNA-dependent RNA polymerase (RdRp) and the exoribonuclease (ExoN). The combination of RdRp and ExoN allow a medium level of nucleotide mutation rate of around 6.677 × 10^–4^ substitutions per site per year, and the nucleotide substitution rate of the spike (S) gene is 8.066 × 10^–5^ substitutions per site per year (1). With the vast number of infected individuals and the virus continuing to surge and mutate, a variety of variants, such as Beta (B.1.351), Delta (B.1.617.2), and Omicron (B.1.1.529), with demonstrated higher transmissibility/infectivity, have evolved. Through evolution, the variant(s) that is the most fit in viral replication/infection and viral-host interaction will evolve into a dominant strain through natural selection.

The human body has a variety of defense mechanisms against the invasion of pathogens (2). One of the first host defenses against upper respiratory pathogens is the respiratory tract barrier. Before reaching the cells, SARS-CoV-2 must overcome several constitutive respiratory defense barriers, including mucus and mucociliary clearance, surfactant proteins, respiratory tract microbiota, and antimicrobial peptides (3). Saliva also contains multiple defense-related proteins that are able to prevent SARS-CoV-2 entry (4). Similarly, defensins are also present in the airway and can block SARS-CoV-2 infection (5). In fact, the defense barriers and early innate immune response that limit the magnitude of initial virus replication are shown to be closely related to the severity of the disease and its outcomes (5–7). A recent study showed that only half of 36 volunteers without previous infection or vaccination were infected after inoculation with WT SARS-CoV-2 (8), suggesting that variance in defense-related components may affect clinical outcome. It has been suggested that the difference in morbidity rate among young adults compared with the older population might be partly related to the difference in their better respiratory tract barriers (4).

Human airway trypsin–like protease (HAT) is a host protease in the respiratory tract, which was initially discovered in the mucoid sputa of patients with chronic airway diseases. It is classified as a member of the type II transmembrane serine proteases, referred to as TMPRSS11D (9–12). Two forms of HAT exist; one is the precursor with a molecular size of 47 kDa and is localized to the ciliated bronchial epithelial cells of airways. The other form of HAT is a mature HAT enzyme that is processed from the precursor and is released into the extracellular milieu (11). This mature form of HAT, with a molecular weight of 27 kDa, is involved in many physiological (e.g., local defense) and pathologic (e.g., asthma, chronic bronchitis, pulmonary fibrosis, influenza virus infection) processes (11, 13–16).

Here, we demonstrate the antiviral effect of mature HAT present in the extracellular milieu on early SARS-CoV-2 isolates and show that the virus has evolved and mutations in the latest Delta and Omicron variants confer resistance to the anti-SARS-CoV-2 effect exerted by HAT. Our data highlight the evolving viral-host interaction, and this viral adaptation may be one of the factors for the enhanced infectivity observed in the Delta and Omicron variants.

## Results

### Anti–SARS-CoV-2 substance from human nasal wash samples.

**Figure 1:**
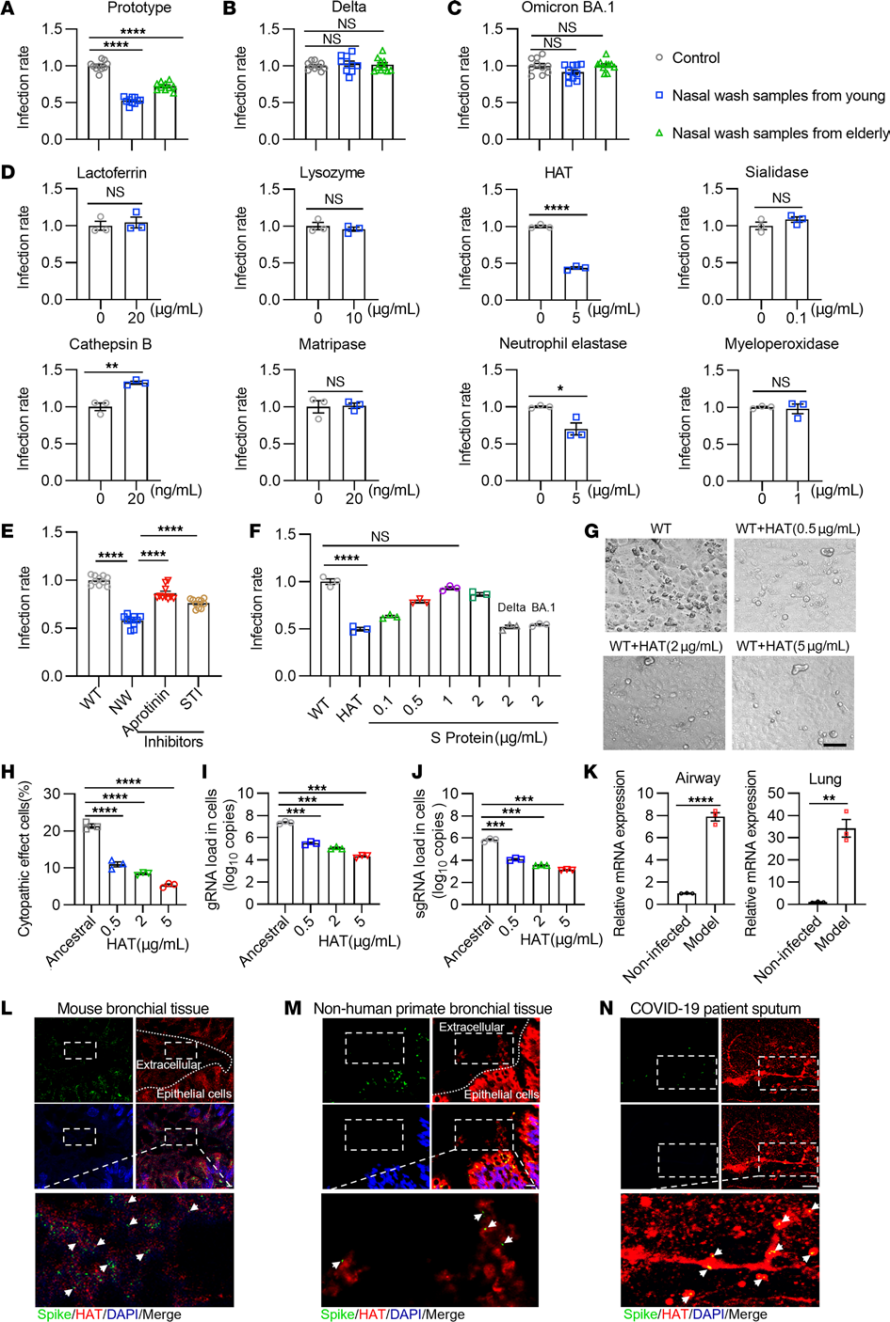
Defense-related component existing in the respiratory tract inhibits infection of ancestral SARS-CoV-2 but not Delta and Omicron variants. (**A**–**C**) Infectivity of WT (**A**), Delta (**B**), and Omicron BA.1 (**C**) pseudoviruses pretreated with nasal wash samples (NW) from young (20–30 years) and old (55–70 years) participants (*n* = 10). (**D**) Infectivity of WT pseudovirus with respiratory tract proteases or proteins (*n* = 3). (**E**) WT pseudovirus treated with NW, with or without HAT inhibitors aprotinin and soybean trypsin inhibitor (STI) (*n* = 10). (**F**) HAT (2 μg/mL) pretreated with spike proteins, incubated with WT pseudovirus, determined infectivity (*n* = 3). (**G** and **H**) Representative images (**G**) and quantification (**H**) of CPE in Vero E6 cells infected with live ancestral viruses, with or without HAT (0.5–5 μg/mL) (*n* = 3). (**I** and **J**) Cell lysates collected 48 hours after infection with live viruses for gRNA (**I**) and sgRNA (**J**) assays (*n* = 3). (**K**) The mRNA levels of *tmprss11d* in trachea (left) and lung (right) tissues in mice infected with SARS-CoV-2 on day 3 after infection (*n* = 3 mice each group). (**L** and **M**) The representative images displayed conjugates (arrow) formed with SARS-CoV-2 spike protein (green) and released trypsin-like protease (red) in bronchial tissues of infected mice (**L**) and primates (**M**). (**N**) The images indicated SARS-CoV-2 particles (green) trapped by filamentous HAT (red) in the sputum of patients with COVID-19. The inset shows magnified conjugates in merged images. Data are representative (**D**, **F**, **G**–**J**, and **K**) of 2 independent experiments with 3 replicates each. Unpaired, 2-tailed Student’s *t* test was performed in **D** and **K**, and 1-way ANOVA analysis followed by Tukey’s multiple comparison post hoc test was conducted in **A**–**C**, **E**, **F**, and **H**–**J**. Scale bars: 200 μm in **G**; 4 μm in **L** and **M**; and 10 μm in **N**. Data are presented as mean values ± SEM in **A**–**K**. *P <0.05; **P < 0.01; ***P < 0.001; ****P < 0.0001.

We obtained nasal wash samples from 20 healthy human volunteers with no previously confirmed COVID-19 infection, stratified into younger and older groups. Nasal wash samples were collected from 20 volunteers with the following age distribution and sex: 5 males and 5 females aged 20–30 years old (the younger group), and 3 males and 7 females aged 55–70 (the older group). The samples were first concentrated by ultrafiltration and then incubation with luciferase-expressing SARS-CoV-2 pseudoviruses containing the ancestral, Delta, or Omicron BA.1 spike proteins for 2 hours. Pseudovirus infectivity was evaluated in a human ACE2 receptor–expressing HEK293T cell-based assay (see Methods). Our data revealed that nasal wash samples from both the younger and older volunteers reduced the infectivity of the WT pseudovirus (Figure 1A), but not those containing the Delta (Figure 1B) or Omicron BA.1 spike proteins (Figure 1C).

We evaluated the anti-SARS-CoV-2 activity of a series of known substances present in the respiratory system in our infectivity assay (Figure 1D). Among the molecules screened, we found that cathepsin B significantly enhanced SARS-CoV-2 infection, while neutrophil elastase significantly inhibited infection, consistent with previous studies (4, 17). Interestingly, we found that HAT (5 μg/mL) significantly inhibited SARS-CoV-2 pseudovirus infection (Figure 1D) and also identified the existence of HAT in nasal wash samples (Supplemental Figure 1; supplemental material available online with this article; https://doi.org/10.1172/JCI174304DS1). We therefore hypothesized that the addition of an inhibitor of HAT to our nasal wash samples would attenuate the anti-SARS-CoV-2 effect. Indeed, incubation of nasal wash samples from young volunteers with aprotinin or soybean trypsin inhibitor (STI), 2 inhibitors of HAT, significantly reduced the anti–SARS-CoV-2 effects of the nasal wash samples (Figure 1E). Mouse airway trypsin-like protease (MAT) is the mouse homolog of HAT, encoded by *Tmprss11d*. Adeno-associated virus (AAV) shRNA platform was then utilized for decreasing the expression of *Tmprss11d* in the local respiratory tissues in mice, and the lavage samples of respiratory tract were incubated with the SARS-CoV-2 pseudovirus. We found the lavage samples from mice indeed can significantly reduce the infectivity of the WT pseudovirus, while this antiviral effect was abolished in the group receiving treatment of AAV5-Tmprss11d-shRNA (Supplemental Figure 2). In addition, we further defined the role of the spike protein and its interaction with HAT; we respectively added free recombinant spike proteins from the WT, Delta, or Omicron strain as antagonists to block the antiviral effect of HAT against WT SARS-CoV-2. The infectivity of WT pseudovirus was significantly recovered with the addition of spike proteins from the WT strain, whereas the addition of spike proteins of Delta and Omicron variants had no observable effect (Figure 1F). To confirm the antiviral activity of mature HAT, a live virus assay was conducted, and the percentages of cells with cytopathogenic changes were determined as previously reported (18). Consistent with the result of the pseudovirus assay, recombinant HAT reduced the percentage of cytopathic effects (CPE) cells infected with the WT strain (Figure 1, G and H). In addition, we showed that HAT significantly decreased the viral entry and replication of WT SARS-CoV-2. Both the viral genomic RNA (gRNA) and subgenomic RNA (sgRNA) in WT virus–infected cells were notably reduced by HAT (Figure 1, I and J).

We next explored the expression of MAT in a murine model of SARS-CoV-2 infection. Airway and lung tissue were obtained from ancestral SARS-CoV-2–infected mice for RNA extraction and analysis by quantitative reverse-transcription PCR (RT-qPCR). Our data revealed that the expression of mouse *Tmprss11d* mRNA in both the lungs and airways were significantly increased in SARS-CoV-2–infected mice (Figure 1K). Fluorescent IHC revealed that MAT was expressed in airway epithelial cells in SARS-CoV-2–infected bronchial tissues of mice (Figure 1L) and nonhuman primates (Figure 1M). The TMPRSS11D staining was also found in the bronchial lumen, representing another form of HAT that released into respiratory tract. Notably, we identified spike protein staining within areas of TMPRSS11D in the extracellular space (Figure 1, L and M). The same phenomenon was also seen in sputum collected from COVID-19 patients (Figure 1N), suggesting SARS-CoV-2 trapping may underlie the mechanism of HAT/TMPRSS11D.

### Delta and Omicron variants showed resistance to the antiviral effect of HAT.

**Figure 2:**
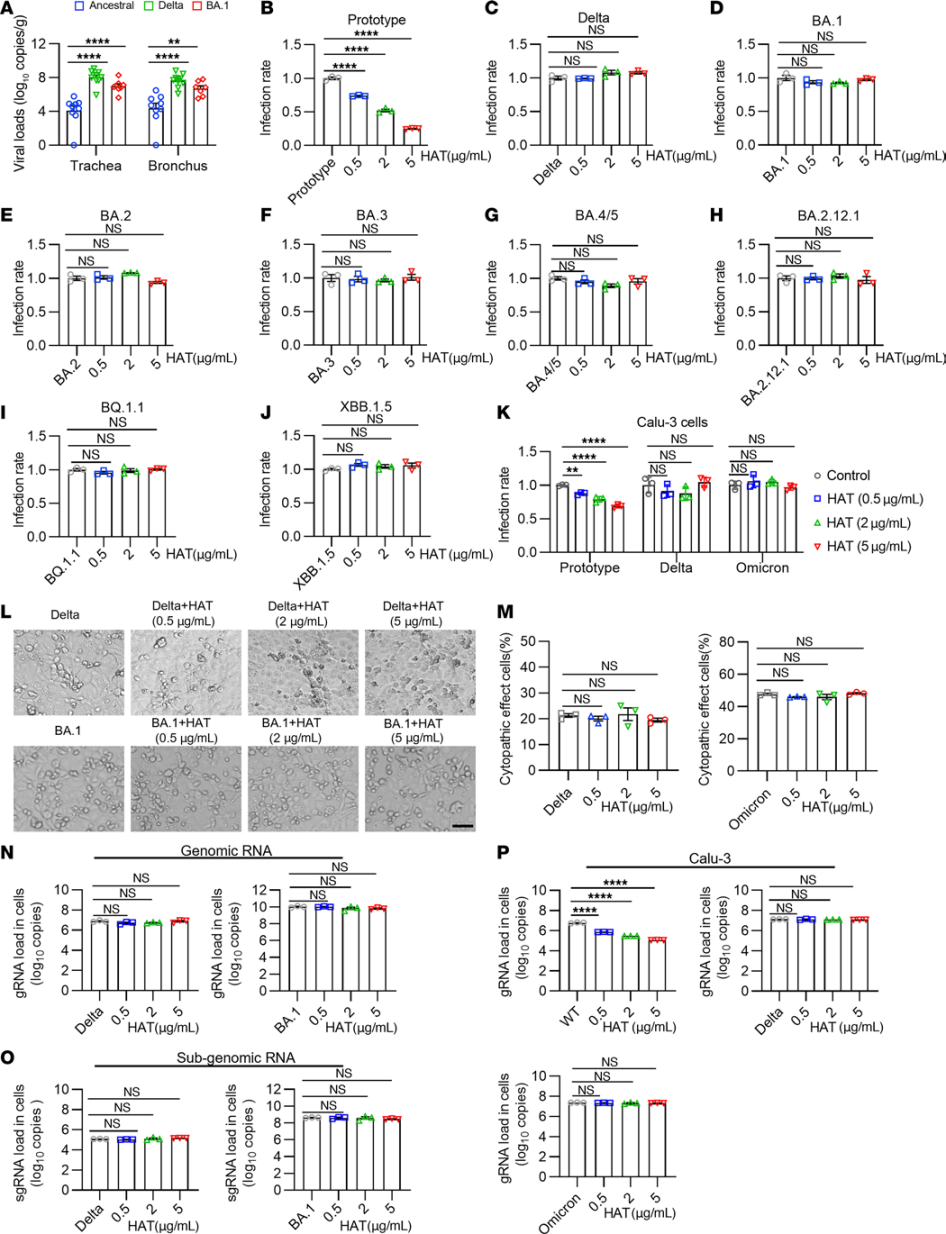
Delta and Omicron variants resist the HAT-induced antiviral effects. (**A**) The viral loads in trachea and bronchus from nonhuman primates challenged with ancestral, Delta, or Omicron BA.1 strains. The data were retrospectively collated from animals in control group that previously used in evaluation of vaccine. All animals were sedated and challenged with 1 × 10^6^ PFU of live viruses via intranasal (0.5 mL) and intratracheal (0.5 mL) routes, and euthanized 7 days after infection for viral loads assay (*n* = 9 nonhuman primates in ancestral virus group, *n* = 10 in Delta group, and *n* = 7 in BA.1 group). (**B**–**J**) The antiviral effects of HAT against WT (**B**), Delta (**C**), and Omicron subvariants BA.1 (**D**), BA.2 (**E**), BA.3 (**F**), BA.4/5 (**G**), BA.2.12.1 (**H**), BQ.1.1 (**I**), and XBB.1.5 (**J**) pseudoviruses were determined. (*n* = 3 each group). (**K**) The infectivity of WT, Delta, and Omicron BA.1 pseudoviruses on TMPRSS2-positive Calu-3 cells in the presence or absence of the HAT (0.5–5 μg/mL) (*n* = 3). (**L** and **M**) The representative pictures (**L**) and quantification analysis (**M**) of cytopathogenic effects (CPE) in Delta and Omicron variant-infected Vero E6 cells (*n* = 3). Scale bars: 200 μm in **L**. (**N** and **O**) Cell lysates from infected Vero E6 cells were collected 48 hours after infection with live viruses for detection of the levels of gRNA (**N**) and sgRNA (**O**) (*n* = 3). (**P**) The gRNA levels of viruses in infected Calu-3 cells with or without preincubation of HAT (*n* = 3). 2-way ANOVA followed by Tukey’s multiple comparison post hoc test was conducted in **A**. 1-way ANOVA followed by Tukey’s multiple comparison post hoc test was conducted in **B**–**K** and **M**–**P**. Data are presented as mean values ± SEM **A**–**K** and **M**–**P**. *P <0.05; **P < 0.01; ****P < 0.0001.

Our data above showed that human nasal wash samples had no protection against the SARS-CoV-2 Delta and Omicron BA.1 variants. We also found higher viral loads in the upper respiratory tracts of nonhuman primates infected with the Delta and Omicron BA.1 variants than with the ancestral variant, raising the possibility that surmounting soluble antiviral molecules such as HAT is a prerequisite for successful infection by these variants (Figure 2A). We therefore explored this apparent HAT resistance in Delta and Omicron variants. We first tested the possibility that addition of exogenous recombinant HAT may overcome this resistance. We found that, while the addition of recombinant HAT (up to 5 μg/mL), could enhance the anti–SARS-CoV-2 effect for the ancestral strain in a dose-dependent manner (Figure 2B), similar additions did not exert any anti–SARS-CoV-2 effect against Delta (Figure 2C), or Omicron BA.1 (Figure 2D) pseudoviruses or live viruses (Figure 2, L and M). Further, we also found that the levels of gRNA and sgRNA were not affected in Delta and Omicron-infected cells after treatment with HAT (Figure 2, N and O). In addition, exogenous recombinant HAT (up to 5 μg/mL) had no antiviral activity against BA.2 (Figure 2E), BA.3 (Figure 2F), BA.4/5 (Figure 2G), BA.2.12.1 (Figure 2H), and the recently rising BQ.1.1 (Figure 2I) and XBB.1.5 (Figure 2J) subvariants. The HEK293T and Vero E6 cells had less expression of transmembrane protease serine 2 (TMPRSS2), while the viral entry into the lung cells is TMPRSS2-dependent (19). We therefore evaluated the antiviral effects of HAT in a TMPRSS2-positive lung Calu-3 cell line. Similar antiviral effects of HAT against WT strain can be observed in TMPRSS2-positive Calu-3 cells by pseudovirus and authentic infection systems, and the Delta and Omicron variants also showed similar resistance to HAT as in 293T/ACE2 (Figure 2, K and P). Thus, our findings indicate that the evolutionary Delta and Omicron variants can resist the anti–SARS-CoV-2 activity of soluble nature of HAT.

### HAT blocked the binding of spike protein to hACE2 receptor and inhibited the membrane fusion process.

**Figure 3:**
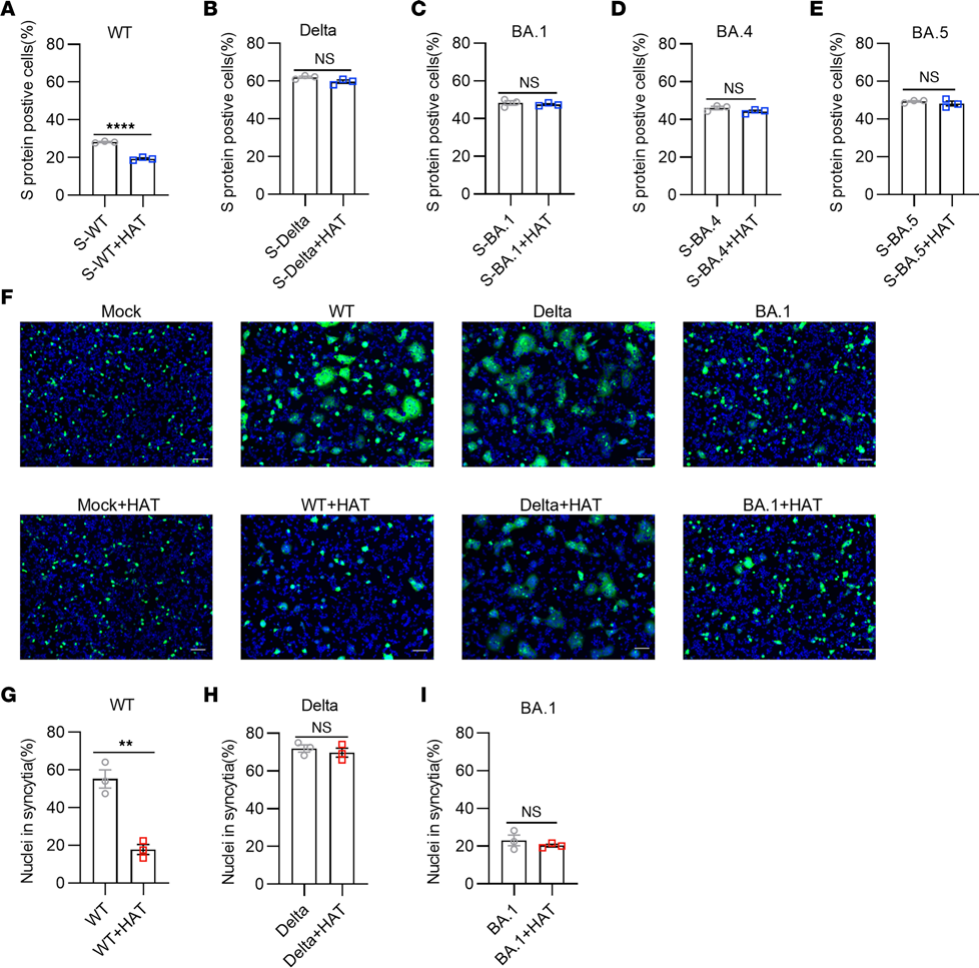
HAT blocked the binding of spike proteins to hACE2 receptor and inhibited the cell-cell membrane fusion process. (**A**–**E**) Flow cytometry analysis of the percentages of spike (S) proteins from WT (**A**), Delta (**B**), and Omicron BA.1 (**C**), BA.4 (**D**), or BA.5 (**E**) strains pretreated with HAT binding to 293T/ACE2 cells (*n* = 3). (**F**–**I**) Effect of HAT on the process of cell-cell fusion. Representative images (**F**) and quantitative analysis (**G**–**I**) of syncytia in the cell–cell fusion. The effector cells expressing spike proteins and EGFP were pretreated with HAT for 2 hours, and the effector cells were then collected and added to target cells that express the hACE2 receptor (*n* = 3). Unpaired, 2-tailed Student’s *t* test was conducted in **A**–**E** and **G**–**I**. Scale bars: 50 μm in **F**. Data are presented as mean values ± SEM. **P < 0.01; ****P < 0.0001.

Cellular entry by SARS-CoV-2 occurs in 2 steps, binding to ACE2 receptor followed by cleavage mediated by membrane-associated serine proteases (e.g., TMPRSS2) (20). We therefore focused on the protective role of HAT on ACE2 receptor recognition and membrane fusion. Human ACE2 receptor–expressing HEK-293T cells were incubated with SARS-CoV-2 spike proteins from the ancestral, Delta, and Omicron BA.1, BA.4, or BA.5 strains, and the percentage of spike protein positive cells was analyzed by flow cytometry. We found that the percentage of ancestral spike protein–positive cells in the presence of HAT was significantly reduced (Figure 3A), suggesting that the binding of the spike protein to the ACE2 receptor was impaired. However, the percentage of Delta (Figure 3B), Omicron BA.1 (Figure 3C), BA.4 (Figure 3D), or BA.5 (Figure 3E) spike protein–positive cells were not altered in the presence of HAT. We also evaluated the formation of syncytia, part of the cytopathic effect caused by SARS-CoV-2 infection. HEK293T cells were transfected with cDNA encoding the ancestral, Delta, or Omicron BA.1 spike proteins, and the formation of syncytia was measured as the number of cells with multiple nuclei (Figure 3F). We found that the percentage of nuclei in syncytia in the ancestral group (Figure 3G) was significantly decreased after pretreatment with HAT, but not in Delta (Figure 3H) and Omicron BA.1 (Figure 3I). These data highlighted the role of HAT in the SARS-CoV-2 infection process and the outcome and implications of the Delta and Omicron variants.

### HAT conferred protection against ancestral SARS-CoV-2 challenge but not Delta and Omicron infection.

**Figure 4:**
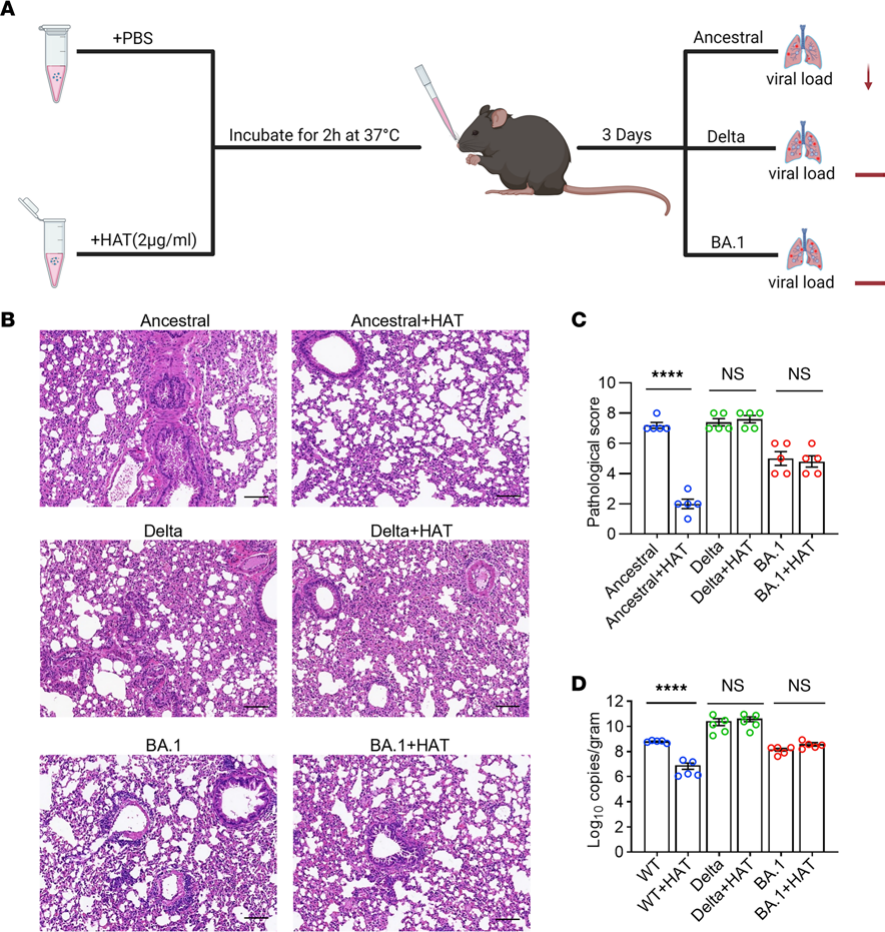
The effect of HAT on the infectivity of ancestral SARS-CoV-2, Delta, and Omicron variants in vivo. (**A**) 5 × 10^4^ PFU of ancestral SARS-CoV-2, Delta, and Omicron (BA.1) variants were preincubated with PBS or 80 ng HAT in a total volume of 40 μL. After incubation at 37°C for 2 hours, 6–8 week-old female transgenic hACE2 (hACE2-KI/NIFDC) mice were intranasally instilled with the mixtures. The lung tissues were collected on day 3 after infection to determine the histopathological changes and the viral loads (*n* = 5 mice each group). (**B** and **C**) Representative images of histopathological changes (**B**) and pathological score (**C**) in the lung tissues in each group. Scale bars represent 100 μm in **B**. (**D**) The levels of gRNA in mouse lung tissues on day 3 after infection in each group were detected by RT-qPCR. 1-way ANOVA followed by Tukey’s multiple comparison post hoc test was conducted in **C** and **D**. Data are presented as mean values ± SEM in **C** and **D**. ****P < 0.0001.

To investigate the antiviral activity of HAT in vivo, 5 × 10^4^ plaque-forming units (PFU) of ancestral, Delta or Omicron viruses were incubated with recombinant HAT before viral challenge in a transgenic hACE2 rodent model (Figure 4A). The lung tissues were collected on day 3 after infection for histopathologic analysis and viral load determination. Histopathologic analysis of lung tissues revealed moderate broncho-interstitial pneumonia in ancestral and Delta strain–infected mice, featuring the infiltration of inflammatory cells, interstitial edema, multifocal areas of consolidation, alveolar septa thickening, and alveolar congestion (Figure 4B). Although the pathogenesis of Omicron is less severe, mild pathological changes could also be observed (Figure 4C). Consistent with in vitro results, pretreatment with HAT significantly attenuated the ancestral strain–induced pathological changes, displaying intact pulmonary alveoli structure, reduced immune cell infiltration, and decreased interstitial edema and necrotic debris (Figure 4, B and C). In addition, the levels of viral gRNA in lung tissue from mice infected with the ancestral strain were remarkably decreased by HAT (Figure 4D). By contrast, no apparent attenuation in pathological changes and viral loads was observed in the Delta or Omicron variants pretreated with the HAT group (Figure 4, B–D). These results further demonstrated that mature HAT plays an important role in the defense against ancestral SARS-CoV-2 infection, while the antiviral effect is disrupted by mutations in Delta and Omicron variants.

### Delta spike protein P681R and Omicron spike protein P681H impaired HAT-mediated spike protein cleavage.

**Figure 5:**
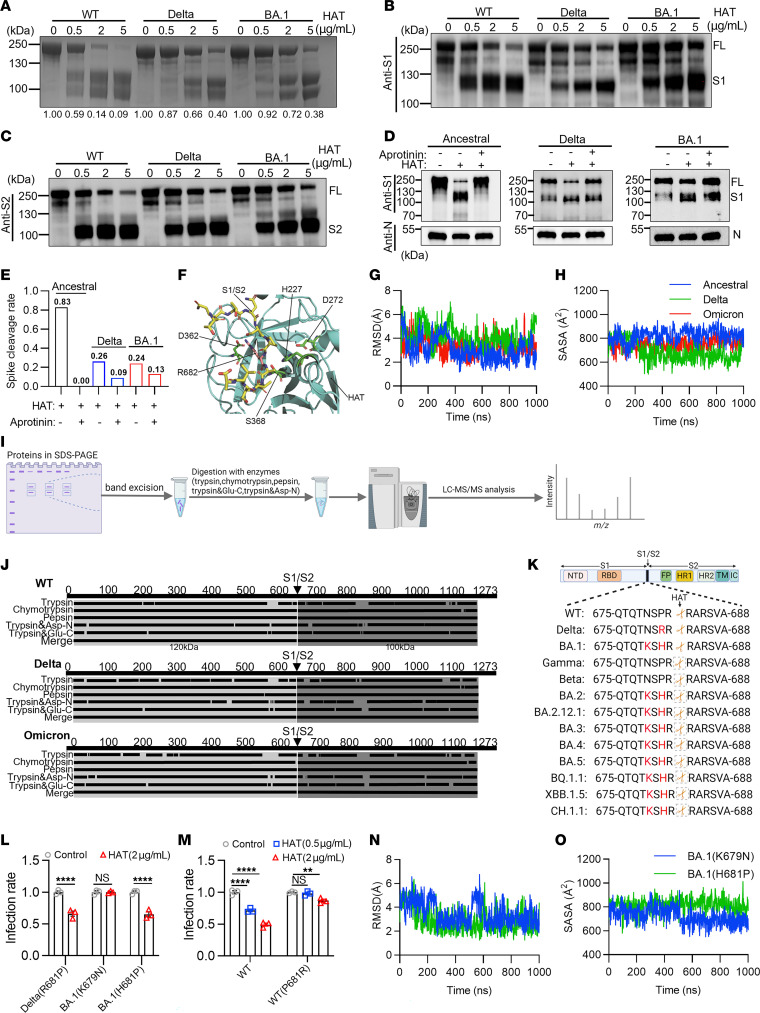
The mutations P681R in Delta and P681H in Omicron spike proteins result in resistance to the HAT antiviral effect. (**A**) Coomassie staining analysis of spike protein cleavage by HAT. Spike protein alone was used as control. Numbers represent normalized band intensities. (**B** and **C**) Western blot assessed spike protein cleavage by HAT using anti-S1(**B**) and anti-S2 (**C**) antibodies. (**D**) Live viruses produced in Vero E6 cells, preincubated with HAT (2 μg/mL) with or without aprotinin, then assayed by Western blot using S1 (top) and nucleocapsid (bottom) antibodies. (**E**) The calculated spike cleavage rate in **D**. (**F**) A molecular model of HAT interacting with SARS-CoV-2 S proteins S1/S2 cleavage site. Proteins are shown in ribbon format, with HAT in cyan and the cleavage site in yellow. Important residues, including the catalytic triad H227, D272, and S368 and salt bridge R682–D362, are shown in stick form. (**G**) RMSD time evolution of SARS-CoV-2 S proteins S1/S2 cleavage site. (**H**) Time evolution of HAT-S1/S2 cleavage site contact area for SARS-CoV-2 S proteins. (**I** and **J**) Cleavage products of spike protein for mass spectrometry: in-gel collection, enzymatic digestion, and analysis. HAT cleaves spike protein at R682 site. (**K**) Diagram of cleavage and surrounding mutation sites in SARS-CoV-2 variants. (**L**) Infectivity of Mut-1 (R681P in Delta), Mut-2 (K679N in BA.1), and Mut-3 (H681P in BA.1) pseudoviruses preincubated with HAT (2 μg/mL) (*n* = 3). (**M**) Infectivity of WT pseudovirus carrying P681R mutation pretreated with or without HAT (0.5–2 μg/mL) (*n* = 3). (**N**) Time evolution of the RMSD of the S1/S2 cleavage site for BA.1 (N679K) or (P681H) mutation. (**O**) Time evolution of contact interface area between HAT and S1/S2 site for BA.1 (N679K) or (P681H) mutation. 2-way ANOVA followed by Šidák’s multiple comparisons test was conducted in **L** and **M**. Data are presented as mean values ± SEM. **P < 0.01; ****P < 0.0001.

We next examined the cleavage activity of HAT against SARS-CoV-2 spike proteins using PAGE and Coomassie staining. Spike proteins from the ancestral, Delta, or Omicron BA.1 strains were incubated with HAT for 2 hours, followed by PAGE and Coomassie staining (Figure 5A). Spike protein in the absence of HAT runs as a single band around 180–250 kDa. In the presence of increasing concentrations of HAT, 2 lower molecular weight bands corresponding to cleaved spike proteins appear in a concentration-dependent manner. It is interesting that, while HAT was ineffective at preventing infection by the Delta and Omicron BA.1 variants, spike protein cleavage occurs, albeit at a lower efficiency, as evidenced by the proportion of full-length spike protein remaining at the highest concentration of HAT (5 μg/mL) tested. This suggests that incomplete cleavage of Delta and Omicron BA.1 spike proteins, rather than noncleavage, was responsible for preserving the infectivity of Delta and Omicron BA.1 SARS-CoV-2 viruses. Interestingly, we also found that the cleavage of Beta and Gamma spike proteins by HAT was better than cleavage of the Delta and Omicron (BA.1) variants (Supplemental Figure 3).

We sought to identify the 2 cleavage bands using antibodies against the S1 and S2 domains. Indeed, the 2 prominent cleavage fragments were the S1 and S2 subunits, with corresponding molecular weights of 120 kDa and 100 kDa, respectively (Figure 5, B and C). We also incubated the authentic viruses and HAT in the presence or absence of aprotinin and found that appearance of the S1 subunit in the ancestral virus group could be inhibited in the presence of aprotinin, indicating that cleavage was indeed mediated by HAT (Figure 5D). The spike proteins of Delta and Omicron BA.1 occurred a spontaneous cleavage, characterized by the detection of bands representing cleavage fragments in the absence of HAT (Figure 5D). Nevertheless, the remaining full-length proteins of Delta and Omicron variants appeared to show significant resistance to HAT (Figure 5D), which is similar to cleavage of spike proteins by nasal wash samples (Supplemental Figure 4). The calculated cleavage rate of spike for ancestral, Delta, and Omicron BA.1 were determined to be 83%, 26%, and 24%, respectively (Figure 5E). As a control, we employed an antibody against the nucleocapsid (N) protein to detect whether the N proteins were cleaved by HAT, and we found no obvious cleavage in the N protein after incubation with HAT (Figure 5D).

We then sought to model the molecular interface between HAT and the spike protein, with the aim of identifying the amino acid residues responsible for impaired cleavage in the Delta and Omicron BA.1 variants. The structure of HAT is unavailable. Therefore, we constructed the predicted protein structure of HAT using AlphaFold (UniProt ID O60235). The predicted HAT structure had a very high level of confidence, with most of the residues having pLDDT scores (21) greater than 90. HAT can be separated into noncatalytic and catalytic regions (11). We only employed the catalytic region (residues 187–418) for further simulation. Considering that HAT shares 56% sequence similarity to porcine trypsin, we expect that both proteins should have similar structure. We aligned the predicted protein structure of HAT to the X-ray crystal structure of porcine trypsin (PDB ID: 1UHB) (Supplemental Figure 5) (22). The root mean square deviation (RMSD) of the 2 aligned structures is 1.49 Å. Both of the structures present a highly conserved catalytic triad with 3 amino acids (H227, D272, and S368 in HAT). The initial binding conformations of the S1/S2 cleavage sites (T676–A688) of the spike protein were constructed manually according to the substrate coordinates in the complex structure 1UHB. We then carried out a microsecond-long all-atom molecular dynamics (MD) simulation to investigate the interaction between HAT and the S1/S2 cleavage site of the spike (S) protein of SARS-CoV-2 (Figure 5F), including WT, Delta, and Omicron variants. The RMSD of HAT for all 3 systems were stabilized at small values, i.e., 2.39 Å, 2.47 Å, and 2.20 Å, respectively (Supplemental Figure 6A). The distances between the C-α atoms of the HAT conserved catalytic triad residues (H227, D272, and S368) (Figure 5F) remained constant throughout the MD for all 3 systems, i.e., around 6.5 Å between residues H227 and D272 (Supplemental Figure 6B) and 8.4 Å between residues H227 and S368 (Supplemental Figure 6C). The distances were consistent with those observed in the X-ray crystal structure of porcine trypsin (PDB ID: 1UHB), providing further confidence in our MD model. Favorable van der Waals and hydrogen bond interactions were observed in the binding interface between the S1/S2 cleavage site and the HAT. The positively charged residue R682 of the S1/S2 cleavage site forms a salt bridge interaction with the negatively charged residue D362 of HAT (Figure 5F).

MD simulation showed that the S1/S2 cleavage sites of Delta and Omicron are more flexible than that of the WT when binding to the HAT. The average RMSD of the S1/S2 cleavage site of the WT was 3.0 Å, which was smaller than that of the Delta and Omicron with 4.2 Å and 3.4 Å, respectively (*P* < 0.05) (Figure 5G). We calculated the average interaction interface area between the S1/S2 cleavage site and the HAT during MD simulation. The contact interface area of the ancestral strain was 826 Å^2^, which is bigger than that of the Delta and Omicron variants, with 689 Å^2^ and 746 Å^2^, respectively (*P* < 0.05) (Figure 5H). The average binding energy of the S1/S2 cleavage site with HAT was –310.0 kcal/mol for the WT spike protein, which is lower and stronger compared with that of Delta and Omicron variants with –282.1 kcal/mol and –290.4 kcal/mol, respectively (*P* < 0.05) (Supplemental Table 1). These results indicated that the S1/S2 cleavage site of the WT forms a more compact and stable binding conformation than that of the Delta and Omicron variants. Compared with the WT, the S1/S2 cleavage site of the Delta and Omicron variants would not be effectively recognized by HAT and show obvious resistance to HAT.

To confirm the cleavage site in the spike protein by HAT, the cleavage fragments were separated by SDS-PAGE, digested with various enzymes, including trypsin, chymotrypsin, pepsin, trypsin with Asp-N and trypsin with Glu-C, and analyzed by mass spectrometry (Figure 5I). The S1 and S2 fragments were both characterized by mass spectrometry with 100% coverage. We found that the cleavage site in the spike protein by HAT was R682 (Figure 5, J and K) (Supplemental Table 2). Then, by searching the genomic database (https://cov-lineages.org/), we noted several mutations (P681R in Delta and N679K and P681H in Omicron subvariants, including BA.1, BA.2, BA.3, BA.2.12.1, BA.4/BA.5, BQ.1.1, XBB.1.5, and CH.1.1) around the cleavage site for Delta and Omicron variants. In contrast, no such amino acid substitutions around R682 were identified in the WT, Beta, and Gamma strains (Figure 5K). Thus, we hypothesized that these mutations might confer the resistance to HAT observed in the Delta and Omicron variants.

We next performed single amino acid mutagenesis by substitution of R681P in Delta (Delta R681P), and K679N and H681P in Omicron BA.1 (BA.1 K679N & BA.1 H681P) pseudoviruses. We found that the Delta R681P and BA.1 H681P pseudoviruses were sensitive to inhibition by HAT in our infectivity assay (Figure 5L and Supplemental Figure 7). By contrast, the infectivity of BA.1 K679N pseudovirus was still resistant to HAT. Notably, single amino acid mutation with P681R based on WT (WT P681R) pseudovirus also exhibited extensive resistance to antiviral effects by HAT compared with prototype pseudovirus (Figure 5M). MD simulation of these amino acid substitutions revealed that the S1/S2 cleavage site with BA.1 H681P formed stable binding conformations with HAT (Figure 5, N and O, and Supplemental Figure 6, D–F). Further, the average binding energy of the S1/S2 cleavage site with HAT was –308.9 kcal/mol for BA.1 H681P mutation, which is lower and stronger compared with that of the BA.1 K679N mutation with –279.6 kcal/mol (*P* < 0.05) (Supplemental Table 3). Our data therefore indicate that position 681 on the spike protein is critical to the activity of HAT.

## Discussion

Our current study showed the role of soluble HAT in the host defense in the upper respiratory tract against SARS-CoV-2. We also showed that the rapid evolution of SARS-CoV-2 has established viral resistance to HAT, as well as identifying the viral resistance mutation. The emergence of resistance against HAT and its enhanced infection in our in vitro and in vivo models highlighted the potential role of these mutations in the observed enhanced infectivity of the Delta and Omicron variants. It also highlighted the importance of early defense in the evolution of the infection.

A variety of substances with antiviral activities, such as immunoglobulins, lysozymes, and defensins, exist in the respiratory tracts and may prevent SARS-CoV-2 invasion (23). Our results showed that HAT plays an important role in the host defense against SARS-CoV-2. It is important for us to emphasize that there may still be other unidentified host factors in the upper respiratory tract with similar functions. A previous study reported that 36 younger volunteers without infection or vaccination were inoculated with SARS-CoV-2, and only 50 percent of participants became infected (8), suggesting that heterogeneity in soluble host defense molecules in the human respiratory tract might contribute to individual susceptibility to COVID-19. In line with previous studies (4), our study showed that nasal wash samples from older individuals were less resistant to the WT SARS-CoV-2 than those obtained from younger individuals (Figure 1A). It is possible that this difference may be related to the reduced amounts of antiviral substances like HAT in the upper respiratory tract with increased age.

The observation of viral evolution that led to resistance to HAT activity was important. Delta and Omicron are both more infectious, based on in vitro and in vivo infectivity studies as well as human population studies (24–28). Our data showing the reduced binding to HAT and the subsequent increase in infection and disease generated in the animal model highlighted the importance of soluble host defense. From an evolutionary perspective, it is not surprising that SARS-CoV-2 has evolved mutations to overcome such immediate host defense mechanisms.

TMPRSS2 is an important host cell protease for viral entry into a target and has been identified as an attractive target for antiviral intervention (19). The serine protease inhibitors Camostat and Nafamostat exhibit antiviral activity against SARS-CoV-2 infection and have been approved for clinical trials (19). Although Omicron showed reduced sensitivity to the antiviral effect of Camostat compared with ancestral and Delta viruses (29), a recent study demonstrated that Omicron maintained dependence on serine proteases for entry throughout the human respiratory tract, and Camostat can still reduce the replication of Omicron (30). Several TMPRSS2-related proteases have been identified for activation of the spike protein, among them, the cell-associated TMPRSS11D (another form of HAT) has been reported to be sensitive to Camostat mesylate (31). Therefore, our current study may provide guidance for the timing of the use of Camostat; the use of Camostat before the viral attachment of host cell membrane may inhibit the activity of mature HAT that is released into the extracellular milieu, thereby reducing its anti–SARS-CoV-2 effect. Nevertheless, such questions should be continued to be investigated in the future studies.

With a combination of mass spectrometry and computational simulation calculations, we were able to determine R682 as the cleavage site in the ancestral spike protein. More importantly, we demonstrated that amino acids around this cleavage site were partly responsible for the reduced activity of HAT on the Delta and Omicron BA.1 spike proteins. We were able to demonstrate that we can reverse the resistance by mutagenesis studies confirming the role of these mutations in their resistance to HAT. Given the large number of mutations found in Omicron variants, it is likely that additional mutations will be implicated in resistance to HAT cleavage. Nevertheless, our findings add significant value in the analysis of future emerging variants, as mutations close to the HAT cleavage site should be considered as potential factors for HAT resistance and may indicate the potential of high infectivity, to be confirmed by infectivity studies and population studies.

It has been reported that the spike protein of SARS-CoV-2 contains additional nucleotides upstream to the single cleavage site 1 forming the PRRAR↓SV sequence (32–36). The unique furin-like cleavage site (FCS) RRAR facilitates the spike protein priming, which might be responsible for its high infectivity and transmissibility (32, 36). More importantly, we have shown that the HAT cleavage site in the spike protein is at R682 rather than the FCS at R685, suggesting that the process by which HAT cleaves the spike protein to block SARS-CoV-2 infection might be distinguished from the S protein priming. A previous study has also demonstrated that FCS can play a role in spike-mediated membrane fusion in the absence of HAT, and it may not be critical for the high fusion capacity of SARS-CoV-2 in the human airway with high levels of HAT (37).

Our study showed that the mutation at P681 is also responsible for the resistance to the antiviral effects of HAT. Notably, the previously and currently prevalent variants, including BA.2.12.1, BA.4, BA.5, BQ.1.1, XBB.1.5, and CH.1.1 Omicron subvariants all harbor mutation 681 (Figure 5K).

We are currently exploring whether a recombinant HAT with higher activity against the Delta and Omicron variants can be engineered. If we are successful, we are contemplating this as a nasal supplement during the endemic/pandemic. It is possible that similar approaches can also be adopted for other viral pathogens.

## Methods

### Sex as a biological variable.

The nasal wash samples were collected from male and female human participants, and only female mice were chosen for animal experiments. Nevertheless, sex was not considered as a biological variable in this study.

### Cell culture.

HEK293T cells were purchased from the American Type Culture Collection (ATCC, CRL-11268). The human ACE2-expressing HEK293T cells (293T/ACE2) were developed in our laboratory. In addition, the Vero E6 (ATCC, CRL-1586) and Calu-3 (ATCC, HTB-55) cells were provided by the Chinese Academy of Medical Sciences and Peking Union Medical College. The cells were maintained in the complete DMEM (Gibco) supplemented with 10% FBS (PAN-Biotech), 100 μg/mL streptomycin and 100 U penicillin (Gibco) at 37°C with 5% CO_2_.

### Collection of nasal wash samples.

Nasal wash samples were collected from 20 healthy adult volunteers by a sterile nasal wash device. The healthy donors included 5 males and 5 females aged 20–30, and 3 males and 7 females aged 55–70. Briefly, 20 mL of saline was flushed into the nasal cavity through the nasal wash device, and the liquid sample was obtained by gravity. To concentrate the proteins in nasal wash samples, 15 mL of the samples were added into the ultrafiltration tube (Millipore), and centrifuged at 2,630 × g for 30 minutes at 4°C through the filter. Finally, 400 μL of the retentate (> 10kDa) was collected and stored at −20°C until use.

### Infection by SARS-CoV-2 pseudoviruses or live viruses.

Different pseudoviruses were used to detect the antiviral effects of HAT on the infectivity of SARS-CoV-2. The luciferase-expressing pseudoviruses of SARS-CoV-2 WT and variants were purchased from Genomeditech. In addition, the pseudoviruses with single amino acid substitutions of the R681P in Delta, and K679N or H681P in Omicron, and P681R in WT pseudoviruses were constructed and generated by Genomeditech. For determination of infectivity, the pseudoviruses were preincubated with nasal wash buffer or recombinant HAT (0.5–5 μg/mL) (R&D Systems, 2695-SE) at 37°C for 2 hours, maintaining the indicated final concentrations in serum-free DMEM containing the indicated inhibitors. The mixtures were added to 293T/ACE2 or Calu-3 cells for 48 hours to express luciferase. The efficiency of viral entry was determined by a multimode microplate reader (PerkinElmer).

To investigate the effect of HAT on the infectivity, live SARS-CoV-2 ancestral, Delta, or Omicron strains (MOI = 0.05) were incubated with HAT in serum-free DMEM at 37°C for 2 hours in a similar way as described above. The mixtures were then added to Vero E6 and Calu-3 cells, respectively. After 6 hours, complete DMEM was added. The cytopathogenic effects in Vero E6 were recorded and calculated after 48 hours. The supernatant was collected and the cells were washed with PBS, followed by cell lysis with Trizol and stored at –80°C. The viral gRNA and sgRNA in Vero E6 and Calu-3 cells were determined by RT–qPCR.

### Enzyme-linked immunosorbent assay.

The existences of HAT in nasal wash samples from human participants were determined using enzyme-linked immunosorbent assay (ELISA) kits (Animalunion Biotechnology Co.,Ltd, LV11027) according to the manufacturer’s instructions. Briefly, the samples were added to a 96-well plate precoated with HAT capture antibodies and incubated for 40 minutes. Then, the plate was washed 3 times and biotinylated detection antibody was added. After incubation for 30 minutes, the plate was washed and avidin-biotin-peroxidase complex (SABC) was added and incubated for 20 minutes. After washing 3 times, 3,3,5,5-tetramethyl biphenyl diamine (TMB) was added to the plate and the plate developed for 10 minutes. Finally, the reaction was stopped and absorbance was measured at 450 nm on a microplate reader.

### Binding of spike proteins from different strains to the cell surface.

Cell surface binding spike proteins was detected by flow cytometry. Biotin-tagged trimeric spike proteins (50 nM) (Sino Biological) from different strains were preincubated with HAT (0.5 μg/mL) at 37°C for 1 hour. The 293T/ACE2 cells were then incubated with the mixture for 20 minutes. The cells were washed with PBS and stained with PE antibiotin antibodies (Biolegend, 409004) at 4°C for 30 minutes. The binding was detected by the Flow Cytometer (ACEA Biosciences) and the results were analyzed with FlowJoV10 software.

### Cell–cell fusion assay.

HEK293T cells cotransfected with a plasmid encoding EGFP (pEGFP-C1) and a vector encoding the SARS-CoV-2 S (WT), S (Delta) or S (Omicron) glycoprotein were used as effector cells. 293T cells expressing human ACE2 receptors on the membrane surface (293T/ACE2) were utilized as target cells. Before the cell–cell fusion assays, the effector cells expressing spike proteins were pretreated with HAT for 2 hours. Then, the effector cells were collected and washed 3 times with PBS. Effector cells and targeted cells were then cocultured at a ratio of approximately 1 SARS-CoV-2 S protein-expressing cell to 1 ACE2 receptor-expressing cell. After further coculture at 37°C for 4 hours, the cells were stained with Hoechst (Beyotime Biotechnology, Hoechst 33342), and syncytium formation between target and effector cells was observed under an inverted fluorescence microscope. 3 fields were randomly selected in each well to count the number of fused and unfused cells.

### Coomassie staining and Western blot.

To confirm the effect of HAT on S proteins cleavage, recombinant spike proteins were produced in 293T cells and obtained from ACRO Biosystems. Spike proteins from different strains (2.5 μg) were incubated with HAT at 37°C for 2 hours. Subsequently, the reactions were stopped by the addition of SDS-loading buffer, and the mixture was boiled at 100°C for 5 minutes. Samples were analyzed by 10% SDS-PAGE and Western blotting. Spike protein cleavage by HAT was quantified by densitometry using ImageJ software. Alternatively, the reaction products were separated by SDS-PAGE with Fastblue (Biosharp) to stain proteins and were analyzed by LC-MS/MS.

To investigate the effect of HAT on live viruses, authentic SARS-CoV-2 ancestral, Delta, or Omicron strains were incubated with HAT (2 μg/mL) in serum-free DMEM at 37°C for 2 hours in the presence or absence of aprotinin and boiled for 5 minutes before analysis using Western blotting. For protein detection, the following antibodies were used: rabbit anti-SARS-CoV-2 S1 polyclonal antibodies (Sino Biological, 40591-T62), rabbit anti-SARS-CoV-2 S2 polyclonal antibodies (Sino Biological, 40590-T62), rabbit anti-SARS-CoV-2 N polyclonal antibodies (Sino Biological, 40143-R001), and horseradish peroxidase–conjugated (HRP-conjugated) anti-rabbit polyclonal antibodies (Thermo Fisher Scientific, 31460).

To calculate the spike cleavage rate in live viruses in Figure 5D, we first obtained the value of band intensities by image J software and normalized the intensities of proteins in each lane followed by calculation of the cleavage rate using the following equation:
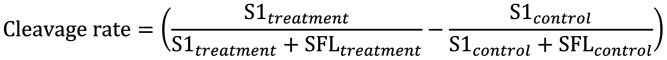


The control group represents only contained authentic viruses, and the sample group contained authentic lives in the presence of HAT or HAT plus aprotinin. S1 is the intensity of S1 band (120 KDa) and SFL is the intensity of full-length spike band (250 KDa).

### Mass spectrometry.

Stained protein bands were excised from the gels and destained, and the proteins in gel were digested by trypsin, chymotrypsin, pepsin, trypsin with Asp-N and trypsin with Glu-C using standard protocols according to the manufacturer’s recommendations. The extracted peptides were desalted and resuspend in 10 μL of buffer A (0.1% formic acid in water) before LC-MS/MS analysis. Then, the peptides were separated on a C18 column (150 μm i.d. × 150 mm, 1.9 μm, 100 Å, Acclaim, Thermo Fisher Scientific) using a 66 minute linear gradient from 4% to 8% B for 2 minutes, from 8% to 28% B for 43 minutes, from 28% to 40% B for 10 minutes, from 40% to 95% B for 1 minute, and from 95% to 95% B for 10 minutes, and the ﬂow rate was set to 600 nL/minute. The samples were analyzed by LC-MS/MS using an EASY NanoLC 1200 system coupled to Q Exactive Hybrid Quadrupole Orbitrap Mass Spectrometer (Thermo Fisher Scientific). The data-dependent acquisition was performed in positive ion mode. The parameters were set as follows for MS1 and MS2 analysis: for MS1, the MS resolution was 70,000 at m/z 200, the automatic gain control (AGC) value was 3 × 10^6^, the maximum IT was 100 milliseconds, and the scan range was 300 to 1,800 m/z. For MS2 scans, the top 20 most intense parent ions were selected with a 3.0 m/z isolation window and fragmented with a normalized collision energy of 28%. MS2 resolution was 17,500, AGC was 1 × 10^5^, maximum IT was 50 milliseconds. The raw MS files were analyzed and searched against the target protein library using Byonic. Carbamidomethyl (C) was set as fix modification. Oxidation (M) and protein N-terminal acetyl were set as variable modifications. Maximum missed cleavage was set to 3. Precursor mass tolerance and fragment mass tolerance for MS1 and MS/MS were set to 20 ppm and 0.02 Da, respectively.

### Molecular modeling of HAT and SARS-CoV-2 S protein S1/S2 cleavage site.

The experimental structure of HAT is unavailable. Therefore, we constructed the predicted protein structure of HAT using the AlphaFold Protein Structure Database with UniProt ID O60235 (https://www.alphafold.ebi.ac.uk/entry/O60235). The model generated had a very high level of confidence, and most of its residues have pLDDT scores (21) greater than 90. The HAT can be separated into noncatalytic and catalytic regions (11). We only employed the catalytic region (residues 187–418) for further simulation. Considering that HAT shares 56% sequence similarity to porcine trypsin, we expect that both proteins should have similar structure. We aligned the predicted protein structure of HAT to the X-ray crystal structure of porcine Trypsin (PDB ID: 1UHB) (Supplemental Figure 5) (22). The root mean square deviation (RMSD) of the 2 aligned structures is 1.49 Å. Both the structures present a highly conserved catalytic triad with 3 amino acids (H227, D272, and S368 in HAT). These verify the reliability of the HAT structure.

The initial binding conformations of the S1/S2 cleavage sites (T676–A688) of the S protein of SARS-CoV-2 (including WT, Delta, and Omicron) were constructed manually according to the substrate coordinates in the complex structure 1UHB. The residue R682 of SARS-CoV-2 S protein forms a highly conserved salt bridge interaction with residue D362 of HAT. There is no steric clash between the atoms of the S1/S2 cleavage site of S protein and the active site atoms of HAT.

The structures of all systems have also been solvated with TIP3P water molecules. The charmm 36 force field (38) was employed. The charge states of protein ionizable groups were normalized corresponding to pH 7.0. Sodium (Na^+^) and chloride (Cl^–^) counter ions were added to ensure the global charge neutrality at a physiological concentration of 0.10 M using Visual MD (VMD). Classical MD simulations were run with NMAD 2.14 (39). The SHAKE algorithm was used to constrain the lengths of all covalent chemical bonds involving hydrogen atoms. The integration time step of the simulations was set to 2 femtoseconds. Nonbonded van der Waals interactions were treated by using a switching function with a cut-off of 12.0 Å. The particle mesh Ewald (PME) algorithm was employed to account for long-range electrostatic interactions. PME is an efficient method for periodic boundary condition. In all cases, the systems were preliminarily minimized using the steepest descents algorithm and then slowly heated from 50 K to 300 K with protein backbone atoms fixed. The systems were then submitted to 10 nanoseconds NPT equilibrations before all-atom MD productions. MD simulations with the whole system relaxed were performed for 1.0 microsecond in isothermal-isobaric NPT ensemble. Constant pressure (P = 1.0 bar) and temperature (T = 300.0 K) was maintained using the Langevin piston coupling algorithm. The structures of proteins extracted from the MD thermodynamic equilibration trajectories were used to calculate the interaction binding energy.

### Mice and experimental protocol.

For the depletion of MAT in vivo, female C57BL/6J mice (6–8 weeks) were grouped into PBS, shRNA scramble, and shRNA tmprss11d groups. The AAV-5 vector was generated after cloning mouse *tmprss11d* shRNA or scrambled fragments into the AAV vector GV478 (Shanghai Genechem Co., Ltd). Following the package, AAV-5 was administrated via intranasal delivery (1 × 10^11^ vector genomes [vg] per mouse) to mice. After 3 weeks, the mice were sacrificed for experiments. The supernatants of respiratory tract lavage samples were collected after centrifugation at 300*g* for 5 minutes and preserved at –80°C to use. Multiple tissues of the respiratory system, including the nasal turbinates, trachea, and lung tissues were harvested and used for the detection of *tmprss11d* expression.

For the challenge of live viruses in vivo, female transgenic hACE2 humanized mice (hACE2-KI/NIFDC) (6–8 weeks) with a C57BL/6J background were provided by the National Institutes for Food and Drug Control (40). The mice were divided into 6 groups: (1) WT SARS-CoV-2, (2) WT SARS-CoV-2 + HAT, (3) Delta variant, (4) Delta variant + HAT, (5) Omicron and (6) Omicron variant + HAT. Briefly, 5 × 10^4^ PFU of WT, Delta, or Omicron strains were incubated with PBS or recombinant HAT (80 ng) in a total volume of 40 μL. After incubation at 37°C for 3 hours, the mixtures were intranasally instilled into transgenic hACE2 mice. On day 3 after challenge, the mice were euthanized for tissue processing. The trachea and lung tissues were collected for assay of viral loads, levels of MAT, histological examination, and immunofluorescence staining. Viral gRNA was measured by the RT-qPCR with the following primer and probe sequences (forward, 5′-GACCCCAAAATCAGCGAAAT-3′; reverse, 5′-TCTGGTTACTGCCAGTTGAATCTG-3′; probe, 5′-FAM-ACGCCGCATTACGTTTGGTGGACC-BHQ1-3′). The mouse *tmprss11d* mRNA levels in trachea and lung tissue were detected with the following primer sequences (forward, GGTACAGCTCCGTAACTCGTG; reverse, GCTGGGAGACATACCCTATGGAT).

### Immunofluorescence staining.

Lung tissues of infected mice and nonhuman primates were used for immunofluorescence (IF) staining. These samples were collected and stored under protocols that were thoroughly reviewed and approved by the Institute of Medical Biology, Chinese Academy of Medical Sciences, Kunming, Yunnan, China, ensuring full ethical and procedural compliance. For assay of HAT in response to the SARS-CoV-2 in humans, the sputum samples were collected from 5 patients with COVID-19 and fixed with 4% PFA on coverslips for 10 minutes. The slides were blocked with blocking buffer for 30 minutes at room temperature and incubated with primary antibodies including chimeric monoclonal anti-SARS-CoV-2 spike RBD antibodies (Sino Biological, 40150-D001) and rabbit polyclonal anti-HAT antibodies (Abcam, ab127031) overnight at 4°C. After 3 washes, the slides were incubated with secondary antibodies including FITC-conjugated goat anti-human IgG (H+L) (Proteintech, SA00003-12) and Alexa Fluor 647-conjugated goat anti-rabbit IgG (H+L) (Cell Signaling Technology, 4414S) at 37°C for 1 hour. Because of the high homology, the anti-HAT antibodies can be cross-reactive to TMPRSS11D of mice and nonhuman primates. Fluorescence images were obtained with an OLYMPUS FV3000 microscope system with OLYMPUS FV31S-SW imaging software. 

### Statistics.

Statistical analyses were performed using Prism 9.0 (GraphPad software). Comparisons between 2 groups were performed using unpaired, 2-tailed, Student’s *t* tests. Multiple groups were compared by using 1- or 2-way ANOVA followed by Tukey’s multiple comparison post hoc test as indicated in each figure legend. P values below 0.05 were considered significant.

### Study approval.

For the operation of nasal wash samples collection, all procedures have been reviewed and approved by the Medical Ethics Committee of Sichuan University, and the approval document number is K2022021. We obtained written informed consent from all volunteers in the research. In addition, all procedures associated with SARS-CoV-2 challenge in mice were reviewed and approved by the Institutional Animal Care and Use Committee of the Institute of Medical Biology, Chinese Academy of Medical Sciences, and performed in the ABSL-4 facility of Kunming National High-level Biosafety Primate Research Center. No nonhuman primates were directly used for experiments in this study. All viral load data and tissue samples used for immunofluorescence staining were sourced from the control group of previous COVID-19 vaccine evaluation studies. The data presented in Figure 2A were retrospectively compiled from the COVID-19 infection animal model sample library of the National Kunming High-level Biosafety Primate Research Center, Institute of Medical Biology, Chinese Academy of Medical Sciences, Kunming, Yunnan, China. As mentioned above, the lung tissue sample used for immunofluorescence staining was also collected from nonhuman primates in the control group. All procedures associated with the SARS-CoV-2 challenge in nonhuman primates were reviewed and approved by the Institutional Animal Care and Use Committee of the Institute of Medical Biology, Chinese Academy of Medical Sciences, Kunming, Yunnan, China (under approval document number DWSP202103003, DWSP202108019, DWSP202203016 and DWSP202203 034) and conducted in the ABSL-4 facility of the Kunming National High-level Biosafety Primate Research Center.

### Data availability.

All data are available in the main text or the supplemental materials. Values associated with the main manuscript and supplemental material, including values for all data points shown in graphs and values behind the reported means are provided in the Supplemental Supporting Data Values file.

## Supplementary Material

Supplemental data

Supplemental data set 1

Supplemental data set 2

Supplemental data set 3

Unedited blot and gel images

Supporting data values

## Version 1 09/17/2024

Electronic publication
