# Supplementary material for: SARS-CoV-2 Delta and Omicron variants resist spike cleavage by human airway trypsin-like protease
Source: J Clin Invest. 2024 Sep 17;134(18):e174304. doi: 10.1172/JCI174304 (PMC11405045; doi:10.1172/JCI174304)
Supplement: Supplemental data [file jci-134-174304-s046.pdf]

Supplementary Information for

SARS-CoV-2 Delta and Omicron variants Resist Spike cleavage by Human Airway Trypsin-like Protease

Correspondence to: xiaweiwei@scu.edu.cn (Editorial corresponding author)

Includes:

Supplemental Figure 1-7

Supplemental Figures

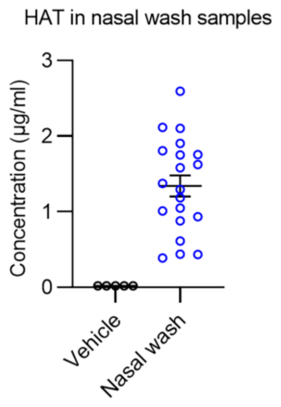

Supplemental Figure 1. The existence of HAT in nasal wash samples were determined by enzyme-linked immunosorbent assay (ELISA). Nash wash samples were obtained from health human participants without history of SARS-CoV-2 infection (n=20 human participants), the vehicle that utilized to flush the nose was used as control group.

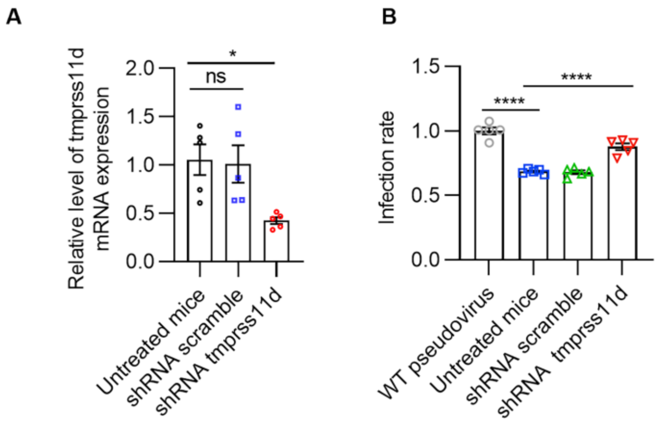

Supplemental Figure 2. C57BL/6 mice were intranasally administrated with PBS, 1×10^{11} vector genomes (vg) of AAV5-Negative control-shRNA (shRNA scramble), and 1×10^{11} vg of AAV5-Tmrpss11d-shRNA (shRNA tmprss11d), respectively (n=5 mice each group). After 3 weeks, the mice were sacrificed and the mRNA expression of *Tmprss11d* in respiratory tissues were determined by RT-qPCR (**A**). The lavage samples of respiratory tract were then collected and incubated with SARS-CoV-2 WT pseudovirus to determine the viral infectivity (**B**). One ANOVA followed by Tukey's multiple comparison post hoc test Data are presented as mean values ± SEM. *P < 0.05, ****P < 0.0001, ns, not significant.

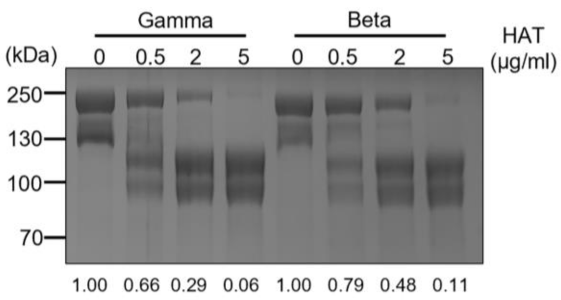

Supplemental Figure 3. Coomassie staining analysis of cleavage of spike proteins from Gamma and Beta by recombinant HAT. Spike protein in the absence of HAT was used as a negative control. The numbers below represent normalized band intensities, and setting the trimeric spike proteins (~250 kDa) in the absence of protease as 1.00.

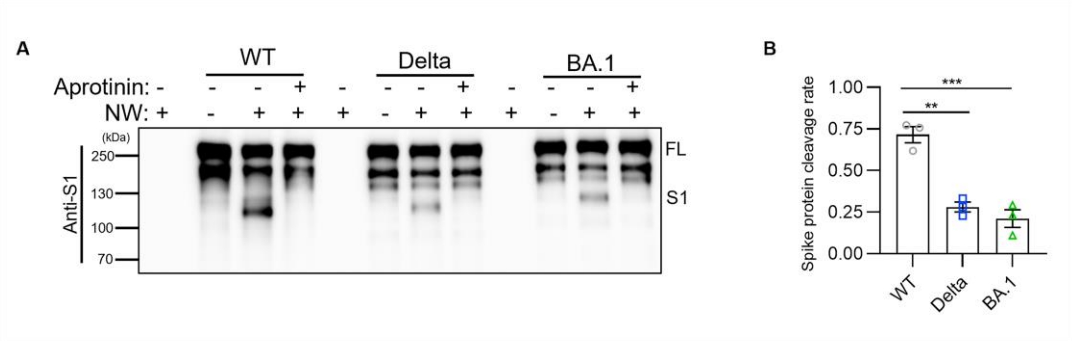

Supplemental Figure 4. Recombinant spike proteins from WT, Delta and Omicron strains were incubated with nasal wash samples from three young human participants, and the cleavage products were determined by western blotting with anti-S1 subunit antibodies. The representative image (**A**) was shown, and the spike cleavage rate of each spike protein were calculated (**B**) (n=3 young human participants). Spike protein in the absence of nasal wash samples was used as a negative control. One ANOVA followed by Tukey's multiple comparison post hoc test Data are presented as mean values ± SEM. $^{**}P < 0.01$; $^{***}P < 0.001$.

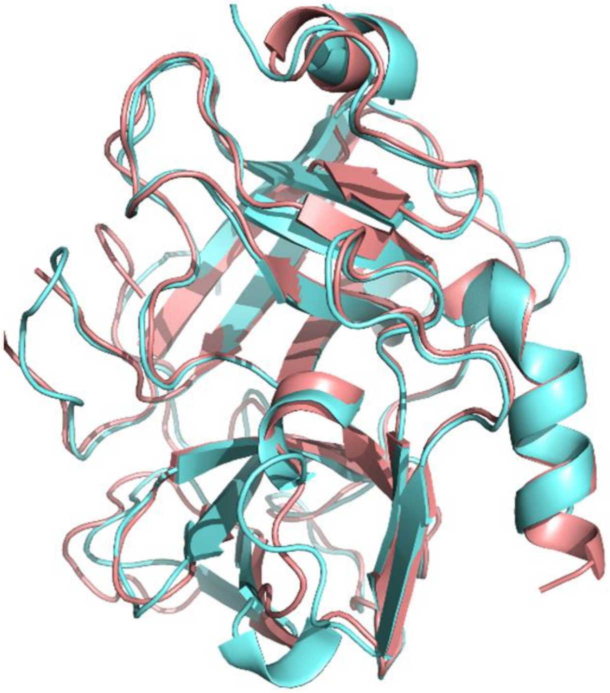

Supplemental Figure 5. The molecular alignment of the predicted protein structure of HAT (shown in cyan) to the X-ray crystal structure of porcine trypsin (PDB ID: 1UHB) (shown in pink). Proteins are represented using the standard ribbon format.

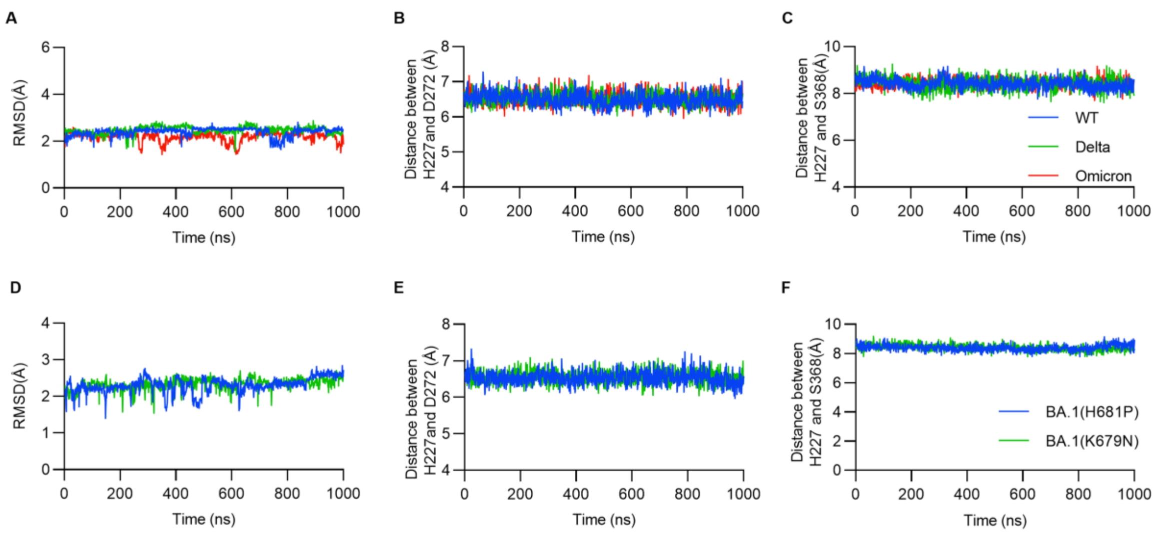

Supplemental Figure 6. (**A**) Time evolution of the HAT's RMSD from the initial structure for the ancestral strain, Delta, and Omicron (BA.1). (**B**) Time evolution of the C-alpha atom distance between H227 and D272 for the ancestral strain, Delta, and Omicron (BA.1). (**C**) Time evolution of the C-alpha atom distance between H227 and S368 for the ancestral strain, Delta, and Omicron. (**D**) Time evolution of the HAT's RMSD from the initial structure for the individual BA.1(N679K) or (P681H) mutation. (**E**) Time evolution of the C-alpha atom distance between H227 and D272 for the individual BA.1(N679K) or (P681H) mutation. (**F**) Time evolution of the C-alpha atom distance between H227 and S368 for the individual BA.1(N679K) or (P681H) mutation.

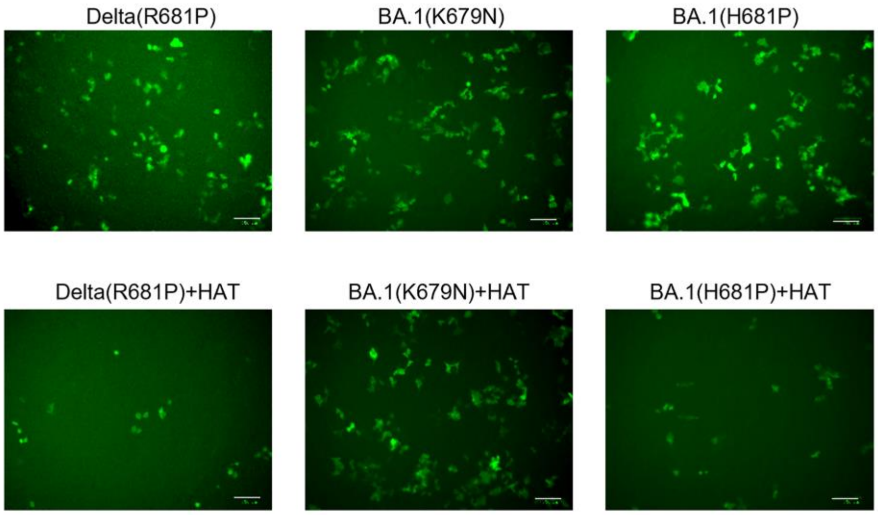

Supplemental Figure 7. The fluorescent images showed that the 293T/ACE2 cells were infected with the Mut-1 (single amino acid substitution of R681P in Delta pseudovirus), Mut-2 (single amino acid substitution of K679N in BA.1 pseudovirus) and Mut-3 (single amino acid substitution of H681P in BA.1 pseudovirus) pseudoviruses preincubated with HAT (2 μg/ml). Scale bars represent 100 μm.
