# Supplementary material for: SARS-CoV-2 Delta and Omicron variants resist spike cleavage by human airway trypsin-like protease
Source: J Clin Invest. 2024 Sep 17;134(18):e174304. doi: 10.1172/JCI174304 (PMC11405045; doi:10.1172/JCI174304)

Original images of gels and western blots

Figure 5A

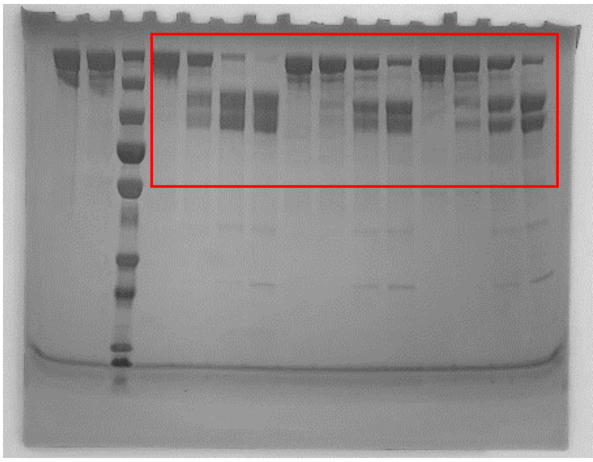

Figure 5B (Anti-Spike S1)

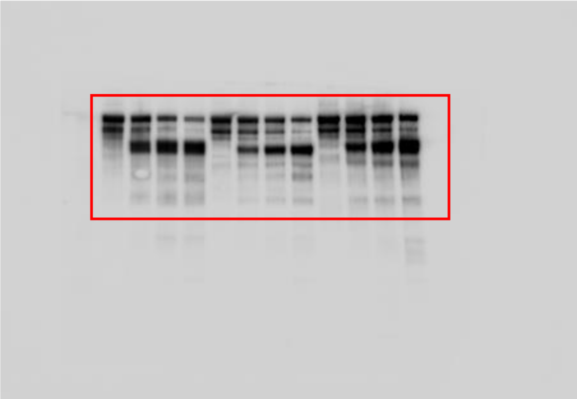

Figure 5C (Anti-Spike S2)

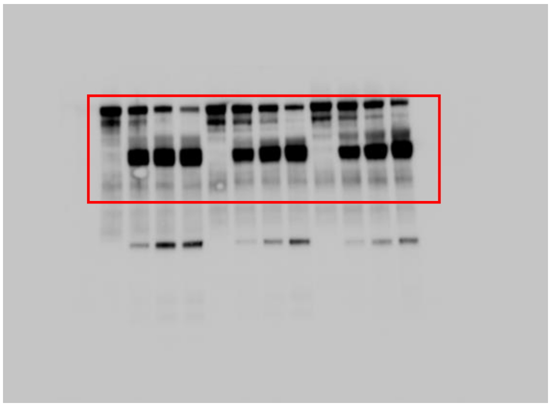

Figure 5D Up (Anti-Spike S1)

Ancestral

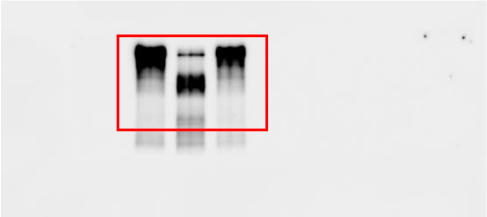

Delta

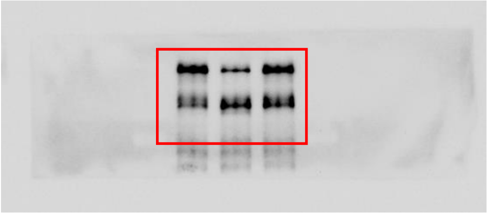

Omicron BA.1

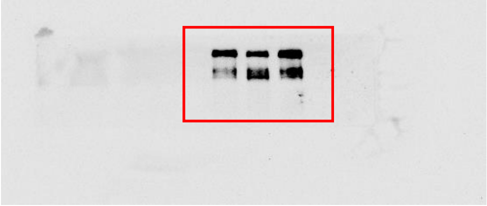

Figure 5D Bottom (Anti-nucleocapsid protein)

Ancestral

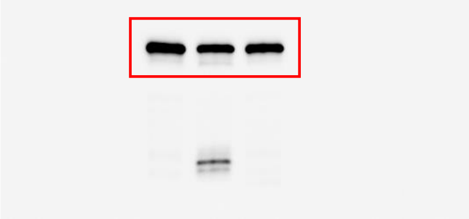

Delta

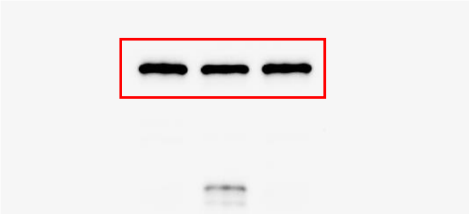

Omicron BA.1

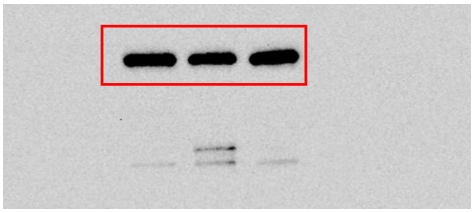

Original images of gels and western blots

Supplementary 3

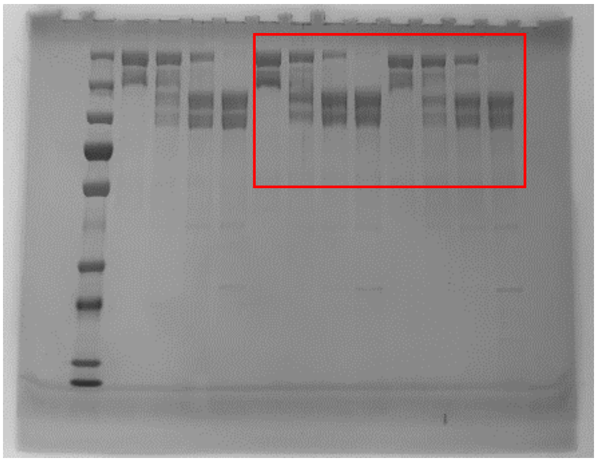

Supplementary 4

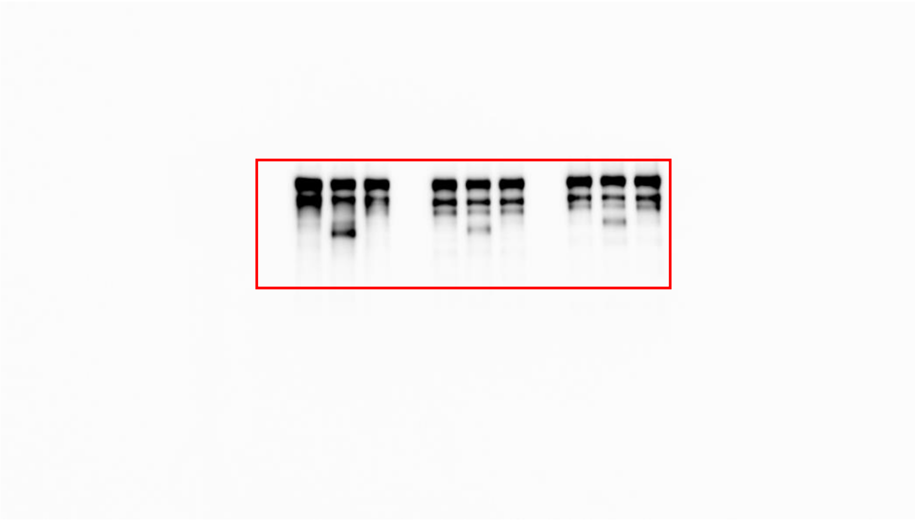

Supplement: Unedited blot and gel images [file jci-134-174304-s050.pdf]
